# Patient Participation in Decision‐Making During Nursing Care: A Relational Autonomy Perspective

**DOI:** 10.1111/jan.70236

**Published:** 2025-09-19

**Authors:** Yin Wang, Lissette Avilés, Colin Chandler

**Affiliations:** ^1^ School of Health in Social Science The University of Edinburgh Edinburgh UK; ^2^ School of Nursing Anhui Medical University Hefei China

**Keywords:** decision‐making, patient autonomy, patient engagement, patient involvement, patient participation, relational autonomy

## Abstract

**Aim:**

To explore patient participation in decision‐making during nursing care experienced by patients with chronic diseases, family members and nurses.

**Design:**

Focused ethnography.

**Methods:**

This study included an 8‐month fieldwork in a Chinese hospital. Fieldnotes from 90 h of participant observation and 30 semi‐structured interviews (10 nurses, 13 patients, three family members, and four with both patients and family members present) were analysed using reflexive thematic analysis.

**Results:**

Patient participation in decision‐making was facilitated in the form of co‐determination, which respected patients' relational autonomy. However, participation required further development or was challenged in the form of unilateral determination, constraining relational autonomy. Interpersonal relationships among nurses, patients and family members played a significant role in promoting patient participation in decision‐making.

**Conclusion:**

A relational autonomy framework was identified to understand patient participation in decision‐making within nursing care. While patient participation is encouraged and autonomy is respected in some situations, paternalistic approaches still persist in clinical practice.

**Implications for the Profession and/or Patient Care:**

Enhancing nurses' awareness of involving patients and family members in decision‐making is needed. The findings highlight the need for better integration and implementation of existing guidelines to support healthcare staff, patients and family members. They also point to the importance of developing culturally relevant frameworks to promote patient participation in decision‐making in nursing care.

**Impact:**

This research provided insight into the experiences of chronically ill patients, family members and nurses regarding patient participation in decision‐making during inpatient nursing care within a non‐Western context. Interpersonal dynamics are highlighted as a key element influencing patient participation.

**Reporting Methods:**

The study is reported using the COREQ checklist.

**Patient or Public Contribution:**

No patient or public contribution.

## 1 Introduction

In contemporary healthcare and nursing practice, patient participation (PP) in decision‐making is increasingly recognised as a central component of person‐centred care, which emphasises viewing patients as active partners rather than passive recipients (McCance and McCormack 2017). PP refers to individuals' active involvement in decisions concerning their treatment and care, and has been associated with a range of benefits, including better health outcomes, greater patient satisfaction, an enhanced sense of empowerment and improved healthcare systems (Birkeland et al. 2022; Halabi et al. 2020).

In the context of chronic illness, where patients often live with and manage their conditions over extended periods, the importance of participation becomes particularly significant. A recent review identified a wide range of strategies to promote patient and family engagement for adults with chronic conditions, spanning direct patient care, health system initiatives and community or policy‐level interventions (Aboumatar et al. 2022). Managing chronic conditions requires not only clinical intervention but also patients' active engagement in ongoing self‐management. Supporting PP can help patients build the confidence, skills and autonomy needed to live with their conditions (Simmons et al. 2014). On the other hand, patients often accumulate experiential knowledge and develop practical self‐care skills through years of managing their conditions. These qualities position them as valuable contributors to care and decision‐making processes, highlighting the importance of fostering the participation of chronically ill patients.

Despite broad support for PP, the current state of participation remains complex. Studies have shown that actual involvement in decision‐making often remains limited, with many patients continuing to occupy a passive role (Lin et al. 2018; Tobiano et al. 2023). At the same time, patient preferences for participation vary considerably. While some individuals express a strong desire to be involved, others may prefer to defer decisions to professionals depending on the situation or the type of decision involved (Bucknall et al. 2019; Noteboom et al. 2021; Ringdal et al. 2017). Moreover, research has revealed that patients' actual experiences of participation do not always align with their stated preferences (Redley et al. 2019; Driever et al. 2022; Wang et al. 2025), highlighting a gap between desired and enacted roles in care processes. These inconsistencies indicate the need for a more nuanced understanding of PP in decision‐making that goes beyond binary notions of active versus passive roles.

These tensions further suggest that participation is not merely an individual choice, but often shaped by patients' capability, contextual constraints and the relationships surrounding care (Buljac‐Samardzic et al. 2022; Cvetanovska et al. 2023). For instance, family members often play an important role in supporting patients and influencing decisions (Keij et al. 2023). To better account for these relational and contextual complexities, this study adopts a relational autonomy perspective, which emphasises how autonomy and participation are constructed through relationships rather than exercised in isolation.

It is also important to acknowledge the terminological ambiguity surrounding PP. Terms such as patient participation, engagement and involvement are often used interchangeably in the literature, although subtle differences have been noted in their semantic, contextual and conceptual meanings (Jerofke‐Owen et al. 2023). In this study, we use ‘patient participation’ as an umbrella term to encompass diverse forms and levels of involvement and engagement, regardless of how participation is framed or enacted in practice. This inclusive usage is especially relevant in the Chinese context, where these English terms often converge into the single Chinese word ‘can yu’. For instance, Zhu (2021) employed the combined phrase ‘patient involvement and participation’ in Chinese hospitals, noting that ‘participation’ implied a higher level of engagement than ‘involvement’. Our terminology choice reflects a similar rationale, which is to capture the full spectrum of patient roles in decision‐making while maintaining linguistic clarity.

## 2 Background

### 2.1 PP in Decision‐Making

Historically, healthcare was dominated by a paternalistic model, where patients were regarded as passive recipients of care who complied with professionals' guidance. From the mid‐20th century onwards, social shifts such as the rise of individual autonomy and the consumer movement catalysed a broader participatory approach to health (Longtin et al. 2010). Within nursing, a move from biomedical to person‐centred philosophies has similarly brought growing attention to PP. Two seminal works of concept analysis (Cahill 1996; Sahlsten et al. 2008) have identified key attributes of PP in nursing care, including an established relationship, shared information, negotiated power and mutual physical or intellectual engagement, which increasingly frame discussions about patient roles in clinical decision‐making.

Shared decision‐making is a widely endorsed approach to PP. It emphasises shared information, respect for patients' values and collaborative decision‐making between patients and healthcare professionals (National Institute for Health and Care Excellence [NICE] 2025). However, implementing shared decision‐making in clinical practice remains challenging in many settings (Driever et al. 2022; Waddell et al. 2021). While shared decision‐making assumes that patients wish to receive relevant information and actively engage in decisions about their care, this assumption does not always align with patient preferences or contextual realities. In certain settings, patients may defer to professional authority due to trust, cultural expectations or a lack of health literacy (Hu et al. 2023; Nuwagaba et al. 2021; Siouta et al. 2023). Moreover, healthcare professionals, including nurses, may prefer to retain decision‐making authority or lack the time, training or institutional support to facilitate shared decision‐making (Liang et al. 2022). These challenges suggest that shared decision‐making may not be universally applicable across all clinical settings or patient populations. Rather than treating shared decision‐making as a one‐size‐fits‐all model, there is a need to explore more context‐sensitive understandings of PP in decision‐making.

Regarding the complexity of PP, earlier studies have highlighted that PP can take various forms and levels. For example, Millard et al. (2006), in a community nursing context, outlined a continuum from fully involving to overt non‐involving, with levels distinguished by the degree of openness, task orientation and responsiveness to patient input. Thompson (2007) explored a taxonomy of patient involvement and participation from professional and patient perspectives respectively, showing that not all patients wish to participate equally, and their preferences can be shaped by illness type, personal traits and relational dynamics. Despite these early insights, recent studies rarely revisit these classifications, suggesting the need to further explore how different forms of participation are experienced and expressed in practice.

While decision‐making in healthcare has been extensively examined, existing literature mainly focuses on medical encounters, such as treatment choices and clinical consultations (Narapaka et al. 2023), with comparatively limited attention to decision‐making embedded in nursing care and everyday support practices. Nurses' roles are often marginalised or limited to low‐stakes or routine decisions. For instance, Galletta et al. (2022) demonstrated that patients perceived nurses paid less attention to involving them in the shared decision‐making process than physicians did. Yet, early studies suggest that patients may prefer greater involvement in relational and day‐to‐day care decisions where nurses are primary actors (Doherty and Doherty 2005).

Unlike medical decisions that typically concern diagnosis, treatment or procedures, nursing decisions often involve patient monitoring, symptom management, care planning and communication about daily care (Siouta et al. 2023). These decisions rely not only on clinical judgement but also on relational interaction and the continuous interpretation of patients' needs. This suggests that the nature of decision‐making in nursing care not only differs in content but also offers unique opportunities for meaningful forms of PP, which are under‐recognised and under‐explored in current research.

Although the term, PP, often appears to focus on the individual patient, a growing body of research highlights the active role that family members play in the process (Hestevik et al. 2020; Jerofke‐Owen and Dahlman 2019; Schjødt et al. 2022). In many cultural settings, PP is not solely the domain of the individual patient, but rather a collective effort involving all caring agents. For instance, an Iranian study found that chronically ill patients, family members and nurses experienced PP in nursing care as a triadic interaction shaped by cultural values of familial interdependence (Soleimani et al. 2010). Mackie et al. (2021) emphasise the value of family involvement and advocate for multi‐level family engagement through promoting relationship‐building, clear communication and transparent information sharing among patients, family members and nurses.

Similarly, in the Chinese context, family involvement in decision‐making is deeply rooted in Confucian traditions, which emphasise caregiving and shared responsibility as core moral duties (Yu and Fan 2007). As a result, it is widely accepted and often expected that family members participate in informed consent and treatment decisions (Badanta et al. 2022). Recognising this, nurses have expressed the need for cultural sensitivity in clinical decision‐making and the importance of respecting family wishes (Chung et al. 2021). Regarding these cultural dynamics, Zhai et al. (2020) proposed a new model encompassing family‐centred care values, rather than directly applying Western‐style shared decision‐making frameworks, to better align with Chinese clinical practice.

These global findings suggest that participation is culturally shaped and relationally embedded. Understanding PP therefore requires not only attention to individual autonomy, but also consideration of the familial and social contexts in which care decisions are made, which is an idea central to the concept of relational autonomy.

### 2.2 Relational Autonomy

PP is deeply rooted in the ethical considerations about patient autonomy, generally denoting the idea that patients have a right to self‐determination and choice regarding the care, support and treatment they receive. This right is nationally and internationally supported by healthcare policies, professional codes of conduct and legislations (Australian Commission on Safety and Quality in Health Care 2025; International Council of Nurses 2021; Lu et al. 2022; Nursing and Midwifery Council 2018).

Yet, the understanding and expression of autonomy can vary across cultural and relational contexts. Traditionally, autonomy in healthcare has been conceptualised through a liberal individualistic lens, emphasising individuals' choices according to their own values and wishes without any interference. The conception of individual autonomy was challenged by feminists, as it was viewed to promote masculine ideals of personhood, which presupposed a conception of the person as atomistic, as ideally self‐sufficient and functioning in a vacuum unaffected by social relationships (Stoljar 2024).

In contrast, relational autonomy, a reconceptualisation of autonomy from a feminist perspective, can offer an alternative framework that acknowledges the social and interpersonal dimensions of human agency. It recognises that people do not live in an asocial vacuum, and values the role of relationships and interpersonal contexts in developing and exercising autonomy (Özdemir 2020). Relational autonomy is thought to capture the interdependent nature of people's lives. In nursing and healthcare contexts, it has been successfully used to examine a wide range of complex care situations, such as the family role in decision‐making, patient autonomy in home care and end‐of‐life care (Gómez‐Vírseda et al. 2020; Ho 2008; Jacobs 2018). In this study, we draw on the concept of relational autonomy as an interpretive lens to capture how patient decision‐making unfolds within interpersonal and institutional dynamics.

This article is part of a broader PhD study that explored PP in inpatient nursing care in China from the perspectives of patients with chronic illness, their family members and nurses (Wang 2025). The larger study identified three primary components of PP: patient self‐care, decision‐making and interdependence with family members. This article focuses specifically on the dimension of decision‐making, offering a more in‐depth analysis and highlighting the relevance of relational autonomy in understanding how PP in decision‐making was enacted in practice (see Appendix S1 for the conceptual framework situating this focus within the broader study).

## 3 The Study

### 3.1 Aim

This study aimed to explore PP in decision‐making experienced by chronically ill patients, family members and nurses in a Chinese hospital.

## 4 Methods

### 4.1 Design

A focused ethnography was employed to explore the cultural and relational practices of decision‐making in nursing care in a Chinese hospital setting (Knoblauch 2005; Wall 2015). The study was informed by a social constructionism approach and symbolic interactionism as its theoretical perspective. Social constructionism holds that knowledge is actively constructed by human beings and shaped by social and historical contexts (Crotty 1998; Porta and Keating 2012). In this view, understandings of PP are not fixed but contextually and temporally shaped.

Symbolic interactionism further informed our lens by emphasising the meaning‐making processes that arise through social interactions (Blumer 1986). Patients, family members and nurses were thus viewed as actors who assign meaning to participation and decisions based on their interactions with one another and the institutional setting.

These theoretical orientations helped Y.W. to explore how decision‐making unfolds in everyday nursing practice in her own society (i.e., China), through attending to situated behaviours, language and cultural norms (Knoblauch 2005).

### 4.2 Study Setting and Recruitment

The study was conducted in a public hospital in Anhui Province, China, with participants recruited from a Neurology Department. This department, which manages over 2200 hospitalisations annually, comprised 47 patient beds across 19 rooms and provided care for patients with chronic cerebrovascular and cardiovascular diseases, and diabetes.

To support recruitment and ensure transparency, study posters were displayed in the Neurology Department. Nurse recruitment for observation and interviews was facilitated through an introductory letter, information sheets and a formal presentation by Y.W. at a monthly nurse meeting, followed by informal conversation with staff. Patients and family members were invited by their primary nurses and Y.W. Nurses initially introduced the study and identified interested individuals, who were then approached by Y.W. for further information and consent.

Participants were primarily recruited through convenience sampling. However, for patients selected for interviews, a combination of convenience and purposive sampling was used, considering gender, age and education level. In the early stages, nurses approached eligible patients without targeting specific demographic characteristics. As data collection progressed, preliminary analysis indicated that these factors (gender, age, education) might influence patients' experiences and interpretations of participation. Purposive sampling was then introduced. For instance, if male participants were overrepresented, nurses were asked to, if possible, invite more eligible female patients. Sampling evolved to maximise diversity and contextual relevance. Nonetheless, all participants willing to participate were included.

### 4.3 Inclusion Criteria

Nurses in the Neurology Department who were registered, with no restrictions on years of work experience, were eligible to participate. The inclusion criteria for patient and family member were as follows: (1) aged 18 or older and able to speak Mandarin; (2) patients whose health condition, as assessed by nurses, was stable enough to participate without risk of deterioration; and (3) family members who were involved in care during the hospital stay, rather than occasional visitors.

An additional inclusion criterion was applied to patients selected for interviews: they had to be hospitalised for at least 3 days. A minimum of 3 days' stay was considered appropriate because, by this time, patients were generally more clinically stable and had settled into the ward environment, enabling them to share their experiences more fully. It also ensured that they had sufficient exposure to the inpatient care process and interaction with nurses, allowing for richer accounts of their participation experiences.

### 4.4 Data Collection

Observation and interviews were conducted concurrently, complementing each other to enhance the depth and richness of data collection (see Appendix S2 for data collection tools in the PhD project). Data collection was conducted aligned with the constructionism stance that views both methods not as neutral data collection techniques, but as co‐constructed processes. Observational fieldnotes were seen as shaped by the researcher's presence and interpretation (Nicholls et al. 2014), while interviews were approached as dynamic spaces where meaning was jointly negotiated and potentially transformative for both interviewer and participant (Yeo et al. 2014).

#### 4.4.1 Participant Observation

Between February and September 2021, a total of 90 h of participant observation across 46 sessions were completed in a Neurology Department. Participant observation significantly facilitated informal discussions, allowing Y.W. to seek further clarification on observed behaviours. The focus of observation evolved over time. Initially, it was broadly descriptive, capturing the overall clinical context. Over time, it became more focused, concentrating on PP practices and eventually shifted to selective observations of key issues emerging from preliminary data analysis (Nicholls et al. 2014).

During fieldwork, Y.W. used a small pocket notebook for immediate note‐taking in the form of keywords, short jottings or direct quotes. These notes captured significant activities, observed behaviours and initial reflections. Full fieldnotes were subsequently expanded and structured into three elements: descriptive accounts of the scene, analytic notes and personal reflections (Nicholls et al. 2014) (see Appendix S2 for template). A pre‐defined format was avoided during real‐time observation due to the unpredictable nature of clinical interactions, though guiding topics from prior fieldwork helped orient each session.

#### 4.4.2 Interviews

A total of 10 nurses, 17 patients and seven family members participated in the interviews. Of these, 10 nurses, 13 patients, and three family members were interviewed individually, while four patient–family dyads were interviewed jointly. In total, 30 interviews were conducted. All invited patients and family members consented to participate, except for one staff member.

Interviews were scheduled individually or jointly based on participants' preferred locations and time within the hospital premises. Most patient and family member participants preferred to be interviewed in their own ward rooms. Occasionally, non‐participants (e.g., other patients or relatives) were present nearby. In such cases, they were informed about the interview and recording and asked for verbal consent. No objections were raised, and confidentiality was emphasised to all present. All participants felt comfortable proceeding under these conditions.

Each participant was interviewed once by Y.W. using a semi‐structured approach (see Table 1 for sample questions). The topic guide (see Appendix S2) was initially developed based on a review of relevant literature and refined through several practice interviews. It was also iteratively informed by insights gained from ongoing participant observation. This reciprocal process allowed the topic guide to remain responsive to context and deepen the exploration of participation as it unfolded in practice. No written notes were taken during interviews to avoid distracting or making participants feel uneasy. All interviews, lasting between 30 and 90 min, were audio‐recorded and transcribed verbatim.

**TABLE 1:** Example interview questions.

Nurse	Have you ever involved patients in decision‐making? If yes, please tell me more details. If not, could you tell me the reasons?
Patient	Do you feel that your voice is heard? How do you communicate your needs and preferences to nurses?
Family member	In what ways do you support or assist patients in being involved in their care?

Sample size was guided by the model of information power (Malterud et al. 2015), which considers study aim, sample specificity, theoretical foundation, dialogue quality and analysis strategy. Early patient interviews yielded limited depth, prompting a larger number of patient interviews to ensure sufficient information power. As data collection progressed, purposive sampling (based on gender, age, education) was introduced to enhance diversity. Family member interviews provided complementary perspectives and tended to be more focused and concise, justifying a smaller sample size. Nurse interviews, by contrast, were often longer, typically lasting over an hour, and were more analytically rich.

After 30 interviews, the research team assessed the data as sufficient to address the study aims, particularly when triangulated with observational data. This judgement was supported by two following months of iterative analysis, during which no major new themes emerged. Thus, no further interviews were deemed necessary.

### 4.5 Data Analysis

NVivo 12 was used for data storage and analysis. Reflexive thematic analysis was chosen for its flexibility and alignment with the study's philosophical position of constructionism (Braun and Clarke 2022). The analysis was conducted in both inductive and abductive ways. In the early stages, we used an inductive orientation to generate descriptive codes from the messy and evolving dataset. As the analysis deepened, we employed an abductive approach, for example, drawing on literature around relational autonomy, to refine codes, make sense of data and increase the abstraction of themes and findings that addressed the research questions (Braun and Clarke 2022).

Data collection and analysis proceeded concurrently throughout fieldwork. Initial coding began during observation and interviews, with Y.W. coding partial data during the process of data generation. Once all data were transcribed, a more systematic coding process was undertaken. The dataset was organised into four source‐based folders in NVivo (fieldnotes, nurse, patient and family member transcripts), each coded sequentially.

The coding across these groups was interrelated. Then, insights from observations were compared and integrated with those from interviews, enabling data source triangulation. For instance, certain behaviours noted in fieldnotes were later discussed or interpreted differently in interviews, and these connections helped develop richer themes. Similarly, codes developed from one participant group informed the analysis of others, supporting a more holistic understanding of PP. Themes were developed across data sources to ensure they reflected multiple perspectives rather than isolated views (see Appendix S3 for a detailed coding process).

To support analytical trustworthiness, several translated transcripts and early coding samples were reviewed by L.A. and C.C. (PhD supervisors), whose feedback helped clarify interpretations and theme development. The entire process was iterative, non‐linear and reflexive (Braun and Clarke 2022).

### 4.6 Ethics Considerations

The study received ethical approval from the Research Ethics Committee at the University of Edinburgh, UK, on February 2021 (NURS056). Additionally, permission to conduct the research was obtained from the hospital's director and nursing administrators. All participants were provided with a written information sheet and a verbal explanation of the study before giving informed consent. Y.W. maintained transparency about her role as a researcher, ensuring that participation was entirely voluntary. Informed consent was obtained, signed and reaffirmed before interviews and observations. Additionally, patients and family members were assured access to support from their primary nurses or physicians during or after the interview, if needed, though none of them requested these offered resources.

### 4.7 Trustworthiness and Reflexivity

Transcripts and findings were not returned to participants for verification. Instead, strategies including prolonged engagement, triangulation, thick description and reflexivity were employed to establish the trustworthiness of this study. Y.W.'s extended immersion in the field, along with data collected from observations and interviews, enhanced the credibility and dependability of the findings. Providing detailed descriptions of participant characteristics and the contextual background further supported the potential transferability of the study to other settings.

In practising reflexivity, Y.W. critically examined how her own positionality, such as age, gender and relationships with participants, influenced the research process. As a registered nurse with prior experience in Chinese hospital settings, she possessed an intrinsic sensitivity to the practice of PP, which informed her observations and interactions. Her shared professional background with the nurses facilitated rapport through daily involvement in their routines. Likewise, for many patient and family member participants, her age was similar to that of their adult children or grandchildren, fostering trust and a sense of benevolence, which are deeply rooted in Confucian values that shape interpersonal relationships in Chinese society (Yu and Fan 2007). This cultural alignment enhanced communication and engagement with participants during the study.

Reflexivity was also maintained throughout the analytical process. For example, the initial research design did not explicitly adopt a relational autonomy lens. When analysing data related to PP in decision‐making, we found that the relational and contextual complexities expressed by participants could not be adequately interpreted through an individualistic autonomy framework. The analytical tension prompted an abductive engagement with existing literature on relational autonomy. This process not only helped articulate the interdependencies within decision‐making practices, but also reflected our interpretive stance as researchers situated within the cultural and clinical field in which the data were generated.

## 5 Findings

**FIGURE 1:**
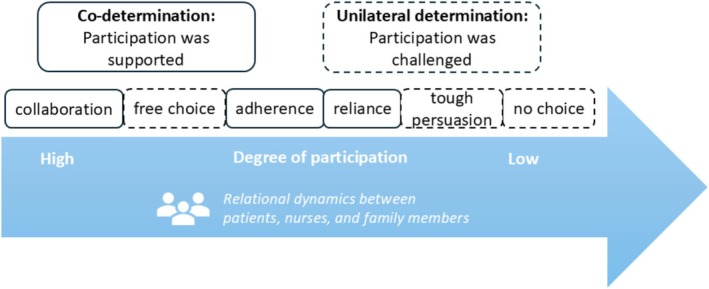
Relational autonomy framework for patient participation decision‐making.

We developed two broad themes of decision‐making that characterised PP in nursing care: co‐determination and unilateral determination (see Figure 1). These patterns were not rigid categories but rather reflected dynamic and situated interactions between patients, family members and nurses. Co‐determination referred to situations where patients' autonomy was respected and participation supported, often through collaboration, reliance and adherence. In contrast, unilateral determination described cases where PP was challenged or constrained, including circumstances of no choice, tough persuasion and free choice. These types did not correspond directly to specific decisions, such as medication or daily activities, but instead illustrated how decisions were made, highlighting the relational dynamics, communication processes and perceived agency across care situations. Participants' characteristics are outlined in Table 2.

**TABLE 2:** Demographic information of participants for interviews.

	Nurses (*n* = 10)		Patients (*n* = 17)		Family members (*n* = 7)	
**Gender**						
Female	10		8		5	
Male	0		9		2	
**Age (years)**						
18–29	7		0		0	
30–39	2		0		0	
40–49	1		5		2	
50–59	0		2		3	
≥ 60	0		10		2	
**Education**						
College	4		5		2	
University	6					
Secondary	0		5		2	
Primary	0		4		2	
No formal education	0		3		1	
	**Professional experience (years)**		**Chronic conditions**		**Kinship to the patient**	
	0–4	7	Cerebrovascular condition	5	Patients' spouse	3
	5–9	1				
	10–14	1	Cerebrovascular and other comorbidities	12	Patients' children	4
	≥ 15	1				

### 5.1 Co‐Determination

#### 5.1.1 Collaboration

In this study, *collaboration* during decision‐making was characterised by patients being offered choices, with nurses and patients mutually considering and respecting each other's perspectives. Collaboration was not simply about allowing patients to decide, but involved continuous negotiation, active listening and a mutual effort to reach shared understanding and agreement. This mutual engagement facilitated shared decision‐making and strengthened the partnership between patients and nurses. For example, one patient noted:

Patient11: I think we should collaborate with nurses. They are the professionals, and we listen to their suggestions. We know our own situations and feelings best. […] I tell them my thoughts and they also listen to me and take it seriously.

Such collaborative decision‐making most often occurred in low‐risk situations, where nurses appeared more willing to empower patients and relinquish control over choices. A typical example was in decisions related to intravenous (IV) therapy.

Nurse09: Some patients refuse to use indwelling needle. I think the indwelling needle is better for them, but they prefer to use the normal one (one off steel needle). Sometimes I just follow their ideas but sometimes I do think there is a need to use the indwelling needle, then I explain more to patients or family members. Most of them accept my suggestions in the end.

In these low‐risk situations, nurses felt more comfortable relinquishing control, as the clinical consequences were perceived as manageable. In contrast, higher risk decisions often triggered stronger professional responsibilities and thus reduced the space for negotiation. For instance, some patients with chronic conditions such as stroke insisted on ambulating independently, despite being assessed at high risk of falling. In such cases, the likelihood of collaboration diminished. Instead, such interactions often developed into other forms of decision‐making, such as tough persuasion or even unilateral actions by patients, as will be explored in later sections.

Beyond direct nurse–patient interactions, collaboration sometimes involved extending communication to physicians, with nurses serving as an important communication bridge. The following fieldnotes illustrate how nurses facilitated patients' voices being heard by medical staff.

Nures08 went to the patient room and asked for the blood glucose measurement. The patient said, ‘I do not want this. I feel well and I do not need to be tested so many times per day’. Nurse08 then replied and left, ‘OK, I will let physicians know’. (Observation29, 1 June 2021)

Later, Nurse08 explained this approach in the interview.

Nurse08: I can understand them because the blood glucose test is also painful for some people. […] Some patients are very experienced, with chronic conditions for years, and they know very well their body. So, I would like to let physicians know their thoughts and reduce the times of blood glucose test per day.

Reaching consensus was not always straightforward. When conflicts or differing opinions arose, nurses employed various strategies to address concerns and manage potential risks to patient safety. These included sharing stories of other patients' experiences, analysing the pros and cons of options, and ensuring patients were fully informed of possible adverse effects associated with their decisions.

Nurse01: At that time, I tell them (patients) some stories about other patients, who made similar choice, causing serious outcomes. It sometimes works. They then made another choice following our suggestions.

These accounts show that collaboration is not only about consensus but also about the willingness of professionals to share decision‐making space when the perceived risks are low. It is important to note that collaboration did not always occur spontaneously but was actively facilitated by nurses through communication strategies, professional judgement and relational engagement.

#### 5.1.2 Reliance and Adherence

In some situations, patients were given choices, but preferred to let nurses or family members decide on their behalf, revealing a form of *reliance* that reflected trust and deference to others' expertise. This was particularly common among older patients, who often felt satisfied with the care received and rarely asked questions. They regarded listening to professionals or family members as the best route to good care.

Patient04: I always said, ‘It depends on you’. They are professionals. It is impossible that they do harm to me.

This reliance was particularly evident in more complex or higher risk clinical decisions, where patients either lacked the confidence to decide or simply preferred not to be directly involved. Instead of disengagement, this reflected intentional trust and a relational approach to care. Family members were often invited by patients as partners in decision‐making.

Patient room. A nurse told a patient of the purpose of the medication she used. The patient just nodded during this process. […] The nurse paused and asked, ‘Does it make sense?’ After a short silence, the patient said, ‘I do not know, nurse. I am so old. How can I know these? My son will come soon. Could you talk to him?’ (Observation33, 9 June 2021)

Nurses also valued the involvement of family members, particularly when communication with older or cognitively limited patients was challenging. Family involvement was seen as a practical strategy to enhance both clarity and efficiency.

Nurse03: It is difficult to communicate with some patients in our department. They are older adults, and sometimes they cannot speak clearly and hear clearly. Or they just cannot understand us. Or they are easy to forget what we have told them. We do not have much time. We usually talk to family members […] and negotiate some issues with them.

Importantly, family members did not merely replace patients in these encounters. In some cases, they played a role as communication bridges, relaying information back to patients and fostering mutual understanding. For example:

A family member (a patient's younger brother) returned to the patient room. The family member appeared to have had a conversation with the nurse. The patient asked his brother about the discussion that had just taken place. Then, the family member explained what the nurse had shared and conveyed the decisions they had made together regarding the patient's care. The patient listened attentively, occasionally nodding. There appeared to be a sense of mutual understanding between the two. (Observation35, 18 June 2021)

Reliance could also merge with more passive forms of *adherence*, in which patients were neither involved nor informed, yet complied without objection. These instances were not characterised by explicit delegation, but rather by default exclusion, particularly during routine procedures such as admission.

Nursing station. During a typical admission session, where initial arrangements for the patient's hospital stay were being discussed. While the patient was present, seated on a chair approximately a meter away, the conversation took place almost entirely between the nurse and the family member (son). The son acted as the primary communicator. This kind of interaction seemed routine, reflecting a pattern where key admission‐related information and decisions are often discussed between family members and nurses, not involving patients directly. (Observation20, 26 April 2021)

Even in such cases, patients themselves often did not perceive this lack of involvement negatively. Instead, they interpreted it as a practical or situational necessity, or as a personal choice shaped by temporary incapacity. In an interview, a patient knew little about the ward rules and his treatment and care plan, but he said:

Patient05: Darling, it is OK. They might talk to my son. You know, on the admission day, I was dizzy and felt unwell. My son was here. It was almost he who talked to nurses and physicians. Or they might have told me, but I forgot it. (laughs) You know, the old people always forget everything!

These scenarios of reliance and adherence suggest that PP does not necessarily require direct involvement in every decision. Rather, patients may participate through intentional delegation or choosing to rely on others in whom they trust. Such relational participation challenges dominant notions of autonomy. It highlights how trust, family relationships and contextual constraints shape everyday forms of participation in care.

### 5.2 Unilateral Determination

#### 5.2.1 Free Choice

When it came to conflicting situations, patients could insist on their own decisions, described here as *free choice*. This often occurred in areas where patients felt confident in managing aspects of their care, including but not limited to medication, symptom monitoring and daily self‐care practices, based on their long‐term experience. For example, a nurse shared her experience with patients and family members requesting care services she considered unnecessary.

Nurse06: […] They (the patient and his daughter) asked us to provide special oral care (brushing patients' teeth with alcohol wipes) for him. I did not know why, and I told them that doing the special oral care did not help to get the high fever down […]. But they still insisted on that.

However, these decisions often went against professional advice, leading to concern or frustration among nurses and family members. Family members described patients as ‘uncooperative’ when they acted according to their own preferences, particularly regarding diet and physical activity.

FamilyP09: He did not exercise, unlike others who would get out of bed and take walks around the ward. […] The nurses told us it would help with his recovery. […] Yesterday, my brother was here, and we both told him, repeatedly. But he just would not listen. He is uncooperative.

At the same time, patients expressed scepticism towards medical recommendations, particularly when these contradicted their own experience of managing chronic conditions. Many patients placed greater trust in their personal knowledge accumulated over years of self‐care, and felt confident in judging what worked for them. From their perspective, the perceived risks of deviating from professional advice were minimal, especially when their symptoms were stable or familiar.

Patient15: I have had hypertension for almost ten years. They (the nurses or physicians) told me to stop taking my medication, but they are wrong. […] They are not professional enough.

While free choice reflected a form of patient autonomy and active involvement in care, it often unfolded without mutual dialogue. On one hand, nurses did not provide comprehensive explanations, and on the other hand, patients were reluctant to seek clarification or negotiate decisions. In some cases, patients made unilateral decisions through concealing behaviours from staff, illustrating a breakdown of trust and collaboration. One field observation showed an example.

Patient room. A nurse spoke to a patient, ‘Why is your IV drip running so fast? Did you adjust it?’ The patient nodded and replied that it was too slow, and he was OK with the increased speed. The nurse slowed down the rate and explained it could be dangerous and asked the patient not to adjust it again. After the nurse left the patient room, the patient talked to his wife, ‘I feel OK with the faster speed’. The wife said, ‘Let's make it faster’. Then he speeded up the IV drip again. (Observation21, 27 April 2021)

Other examples included patients independently taking oral medications, or leaving the ward for a walk without informing staff. These free‐choice behaviours, though based on their perceived expertise, at times posed risks to safety and compromised care quality. In interviews, nurses often described such actions as ‘patients participating too much’, revealing a sense of frustration and moral tension as they struggled to balance respect for autonomy with their professional obligation to ensure patient well‐being.

Nurse02: We are also concerned about this. […] They sometimes pretend that they comply with us. But when we go away, they just do what they want. There is nothing we can do. We are also helpless.

These accounts revealed that free choice often occurred without mutual engagement or shared understanding. Such participation was frequently accompanied by tensions among patients, nurses and family members. While patients regarded their decisions as legitimate expressions of autonomy, others involved in care often struggled to reconcile these choices with their professional or familial responsibilities. Thus, although patients appeared to participate by exercising autonomy, free choice in these cases disrupted opportunities for meaningful dialogue and undermined the development of collaborative care.

#### 5.2.2 Tough Persuasion and No Choice

In some situations, nurses sought to persuade patients to comply when opinions diverged. Nurses often exercised more control over decisions they perceived as clinically significant or high risk, such as some IV therapy procedures or physical activity. However, persuasion was not presented as open dialogue, but as a means to secure compliance, sometimes by invoking medical authority.

Nurse01: If they (patients or family members) do not listen, we communicate with them firstly, letting them know the relevant risks. But if it still does not work, we will say, ‘If you insist on that, we will let physicians know’. Then they will immediately say, ‘OK, OK, I will do what you said’. (laughs). […] Though this was not their own intention, and they may be unhappy about it.

Similarly, patients sometimes appeared to acquiesce not because they were convinced, but because they felt unable to challenge the nurse's stance or feared confrontation.

Patient15: The nurse said I should not walk so much, but I felt fine. I know myself best. […] For nurses, I know why they do not give the permission. If I have a fall, they will be in a trouble. They exaggerate the situation. But I know I can get out of the bed. […] Still, she looked serious, and I did not want to argue. So I just stayed in bed.

We interpreted these practices as *tough persuasion*, distinct from collaborative negotiation. Nurses often presented decisions as fixed, grounded in clinical correctness and safety. Although patients were not overtly excluded, their role was often reduced to reluctant compliance, indicating a subtle form of paternalism.

In some cases, patients were entirely excluded from decision‐making and not even informed. Unlike reliance or adherence, where patients actively deferred control, these *no choice* scenarios often left them confused or dissatisfied. For instance, as shown in the fieldnotes below, the patient appeared unaware of what was being done or why.

Patient room. Nurse02 provided oxygen therapy to a patient and explained the need to the daughter. The patient remained silent. A few minutes later, Nurse04 entered and asked about the therapy. The patient suddenly said, ‘What is going on? How can I know? Why am I here with this device?’ The daughter stood by and said nothing. (Observation41, 24 June 2021)

In this case, the daughter, though informed, remained silent and did not relay the information to the patient, leaving him in uncertainty and disempowerment. These instances reflect a paternalistic approach, in which family members were positioned as proxy decision‐makers, often by default. Nurses directed explanations and justifications towards family members, bypassing patients entirely. However, family members did not necessarily act as communicative bridges. For example, a son caring for his father noted:

Family member02: I think it is better not to tell him too much, he tends to overthink. But, honestly, I am not sure how to explain it either.

Patients' attitudes in these situations were often ambiguous and ambivalent. Some expressed dissatisfaction, while others accepted being shielded from upsetting truths. As one patient explained:

Patient11: I know they did not tell me the real situation. I can feel that. But I understand them. They were worried that I would feel anxious if I knew the truth.

Patients' emotional vulnerability was used to justify their exclusion, especially in decisions related to diagnosis or serious illness. However, such protective practices, even when well‐intentioned, might bypass patient autonomy. Decisions were made without checking whether patients wanted to know or participate. These moments revealed how, in the name of care and protection, participation was not only limited but fundamentally undermined.

## 6 Discussion

Our study identified two overarching types of PP in nursing decision‐making, co‐determination and unilateral determination, each comprising three distinct forms (Figure 1). We found that PP is highly situational, existing on a spectrum shaped by three interrelated elements: the degree of patient involvement, ranging from active decision‐makers to passive recipients; the nature of decisions, spanning from everyday, low‐risk choices to complex, high‐risk medical interventions; and the relational dynamics between patients, nurses and family members, varying from trust and openness to hierarchical control, protection or even exclusion. Across this spectrum, participation is expressed, supported or constrained in distinct ways. This variation can be best interpreted through the lens of relational autonomy, which emphasises that individuals' agency is fundamentally shaped and exercised within interpersonal and social relationships. Significantly, our findings suggest that PP is not only about respecting patient autonomy but also about acknowledging patients' interdependence and ensuring accountability in care decisions.

At the most participatory end of the spectrum, collaboration involved shared decision‐making through open dialogue, where nurses actively engaged patients, respected their views and negotiated plans together. This reflects the ideal of PP as commonly described in the literature, where participation is co‐constructed between professionals and patients (Thompson 2007). It also aligns closely with relational autonomy, as patients' preferences were heard and integrated through reciprocal relationships, rather than isolated, individual decisions. Importantly, collaboration was more likely to occur in situations where patient perspectives were considered valuable and the perceived clinical risks were manageable (Tobiano et al. 2016).

Free choice represents patients making decisions independently, often in ways that contradict professional advice and occur without shared dialogue. While this reflects a form of autonomy grounded in patients' confidence from long‐term experience, nurses frequently viewed it as problematic, particularly when such choices seemed to compromise safety. This highlights that autonomy alone does not equate to meaningful participation.

Free choice also reveals a tension between respecting patient autonomy and upholding professional responsibility. Respect for autonomy should not overshadow other ethical principles, such as beneficence (Cheraghi et al. 2023). From a relational autonomy perspective, genuine participation occurs within networks of relationships and interdependencies, requiring not only deference to patients' preferences but also fostering mutual accountability (Greaney and O'Mathúna 2017). Therefore, free choice should not be seen as the endpoint of participation, but often signals incomplete or disrupted participation. Promoting further participation through negotiation, such as the model to enhance person‐centred communication (Kwame and Petrucka 2021), opens the way for more co‐determined decision‐making processes.

In contrast to more active forms of participation, reliance and adherence represented situations where patients did not directly engage in decision‐making but instead entrusted nurses or family members with this responsibility. This delegation often reflected deep trust and deference, especially among older patients or those facing complex, high‐risk decisions. Despite the apparent lack of direct involvement, these forms of participation were still recognised as expressions of PP, rooted in relational autonomy. Rather than diminishing autonomy, these cases illustrated how autonomy can be supported and exercised through interdependent relationships that enable patients to navigate care decisions appropriately (Cole et al. 2014; Sullivan and Niker 2018). Particularly in the context of chronic illness, where patients' autonomy may fluctuate (Killackey et al. 2019), reliance and adherence highlight that choosing to delegate is itself a meaningful form of agency.

Our findings align with studies (Khosravi et al. 2025) in which some patients preferred forms of reliance or adherence in their care, though sometimes named differently, as welcomed paternalism (Moser et al. 2006) or supportive part of paternalistic care (Pourgholam et al. 2022). These patterns reflect the concept of maternalism (Sullivan and Niker 2018), especially relevant in some cultural contexts (e.g., China), where decision‐making often occurs within caring relationships aimed at preserving patient welfare. Maternalism distinguishes itself from traditional paternalism by occurring within relationships of mutual trust, where the maternalist, such as family members or nurses, deeply understand the patient's needs and ensure that interventions support, rather than undermine, autonomy. This evolving understanding of autonomy calls for a reconceptualisation of paternalism (Sullivan and Niker 2018), recognising supportive, trust‐based delegation as an integral part of meaningful PP.

Tough persuasion marked a turning point in the participation spectrum. Patients were not entirely excluded but were pressured into agreement through asymmetrical power dynamics, particularly in high‐stakes or safety‐critical situations. At the most restrictive end, no choice involved the complete exclusion of patients from decisions, often justified by a desire to protect them emotionally and maintain harmony (Wang and Nolan 2016). Both tough persuasion and no choice reflect paternalistic practices that constrain PP in decision‐making. Although often well‐intentioned, such approaches diminish patient agency and undermine relational autonomy by bypassing mutual respect and silencing patients' voices. Existing literature confirms that paternalism remains prevalent in healthcare and presents a significant barrier to genuine PP (Lazcano‐Ponce et al. 2020; von Humboldt et al. 2024). Addressing this requires increasing nurses' awareness of patient autonomy and promoting supportive caregiving behaviours that foster shared decision‐making (Pourgholam et al. 2022).

Our findings highlight the importance of cultural context in shaping clinical decision‐making. Hawley and Morris (2017) found that patients from diverse racial, ethnic or cultural backgrounds often appraise their decision‐making experiences less positively than white, US‐born patients. They thus called for more culturally appropriate approaches to shared decision‐making. In our study, the prominent role of family members rooted in Chinese cultural traditions was highlighted. The family, rather than the individual, is often seen as the core unit of decision‐making, shaped by Confucian traditions that prioritise filial piety and collective responsibility (Lin et al. 2013; Wang 2015).

In this context, patients often saw family involvement as supportive rather than contradictory to autonomy. Practices that might be labelled as paternalistic in Western settings may instead reflect relational autonomy in East Asian cultures. However, our findings also revealed tensions, particularly in no‐choice scenarios, where family members dominated decisions and patients were effectively excluded. These dynamics resonate with what Zhai et al. (2020) described as the ambivalence of family involvement: while culturally valued, it may at times undermine informed consent and individual agency.

This tension emphasises the need to balance all four bioethical principles, that is, autonomy, beneficence, non‐maleficence and justice, rather than prioritising one over others (Tsai 2005). In many cases, professionals and family members focused on protecting or helping the patient, even if that meant withholding information. While such actions may lead to short‐term benefits, they risk eroding patient trust and long‐term engagement. Therefore, promoting PP requires not only awareness of cultural norms but also ethical reflexivity in practice.

These insights are not unique to China. Cross‐cultural studies have acknowledged the important role of family members in decision‐making in various settings, such as India (Lepping and Raveesh 2014) and Croatia (Murgic et al. 2015). Additionally, the Confucian emphasis on relational personhood aligns with feminist and communitarian ethics in the West, which similarly recognise that autonomy is embedded in care, duty and social connection (Tsai 2005). Wang (2015) also argued that Confucian ethics was not merely a culture‐specific version of autonomy. Thus, the relational autonomy framework we propose is not culturally exclusive but offers a universal lens helping to understand how PP can be better supported across diverse healthcare contexts.

### 6.1 Limitations

This study was conducted in a public hospital in China during the global COVID‐19 pandemic. The findings may be contextually bound to the Chinese healthcare system and specific to the inpatient setting, and may not be directly transferable to other cultural or clinical contexts. In addition, the pandemic posed constraints on patient recruitment. Only patients with cardiovascular and cerebrovascular diseases and diabetes were included, which may not fully represent the broader population of individuals with chronic illnesses.

## 7 Conclusions and Implications

This study introduced a relational autonomy framework to understand PP in nursing decision‐making, particularly within the contexts where interpersonal relationships and cultural norms play a significant role. Our findings highlight that PP is a dynamic and context‐sensitive process, shaped not only by patients' preferences but also by how trust, responsibility and emotional concerns are navigated between patients, nurses and family members, which broadens the current understandings of PP.

In healthcare systems where policy frameworks for PP remain limited, such as in China (Zhu and Sui 2024), there is a need for more explicit, context‐sensitive policies. These could include recognising the realities of decision‐making involving patients, family members and professionals such as nurses. For example, in the United Kingdom, the shared decision‐making guideline NG197 (NICE 2021) could be helpful. However, a critical evaluation of how these can be interpreted and better implemented in practice is needed. Equally, greater attention is suggested to ensure that such guidelines acknowledge relational dimensions of autonomy and accommodate culturally embedded practices.

Our proposed framework can inform nurse education and clinical practice by encouraging critical reflection on relational dynamics in decision‐making. It can help practitioners recognise when participation is meaningful versus tokenistic, and how to balance autonomy with cultural values. Educational efforts should also promote cultural sensitivity and inclusive communication, enabling nurses to engage more significantly with both patients and family members.

## Author Contributions

Y.W., L.A. and C.C. made substantial contributions to conception and design, or acquisition of data, or analysis and interpretation of data; were involved in drafting the manuscript or revising it critically for important intellectual content; gave final approval of the version to be published. Each author should have participated sufficiently in the work to take public responsibility for appropriate portions of the content and agreed to be accountable for all aspects of the work in ensuring that questions related to the accuracy or integrity of any part of the work are appropriately investigated and resolved.

## Ethics Statement

The authors confirmed that any data utilised in the submitted manuscript have been lawfully acquired in accordance with The Nagoya Protocol on Access to Genetic Resources and the Fair and Equitable Sharing of Benefits Arising from Their Utilization to the Convention on Biological Diversity. The relevant fieldwork permission was obtained by the Research Ethics Committee at the University of Edinburgh, UK (NURS056).

## Conflicts of Interest

The authors declare no conflicts of interest.

## Supporting information


**Appendices S1–S3:** jan70236‐sup‐0001‐AppendicesS1‐S3.docx.

**Appendix S4:** jan70236‐sup‐0002‐AppendixS4.pdf.

## Data Availability

The anonymised data that support the findings of this study are available on request from the corresponding author. The data are not publicly available due to privacy or ethical restrictions. Additionally, access is inherently subject to restrictions outlined in the participant information sheets and consent forms. We appreciate your interest in our data and apologies for any unavailability and inconvenience this may cause. The study was reviewed and approved by the Research Ethics Committee at the University of Edinburgh, which may also be contacted for further information at hiss.ethics@ed.ac.uk.
