# Supplementary material for: Patient Participation in Decision‐Making During Nursing Care: A Relational Autonomy Perspective
Source: J Adv Nurs. 2025 Sep 19;82(6):6401–15. doi: 10.1111/jan.70236 (PMC13176680; doi:10.1111/jan.70236)

Supplementary files

# Supplementary 1. The PP conceptual model developed from the PhD project

In the PhD study, PP in nursing care was perceived and experienced as encompassing patient self-care and **decision-making processes**. These processes involved the active participation of family members and support from nurses, and were influenced by individual capability, responsibility, and willingness. The conceptual model (see **Figure 1.1**) was developed, illustrating three main messages from the study: (1) components of PP, (2) facilitators and barriers to PP, and (3) essence of PP.

This paper focuses on the decision-making component, as highlighted in the red box of the **Figure 1.1**.

**Figure 1.1 The conceptual model of PP in nursing care**


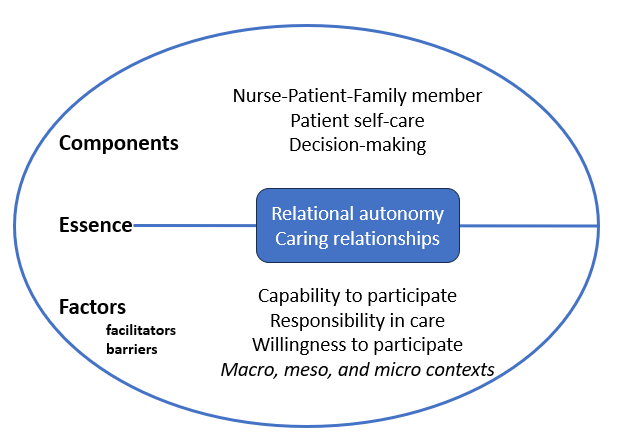


# Supplementary 2. Data collection tools in the PhD project

# 2.1 Recording observational data: fieldnotes template

Following Nicholls et al. (2014), I included three key elements in the fieldnotes: detailed descriptions, analytic notes and observer comments about the setting, and subjective reflections. A template for fieldnotes is provided in **Table 2.1**, with some examples in italics.

**Table 2.1 Fieldnotes template**

| **Session No.....**  **Date: ......**  **Start time: .......... End time: ............Duration: ...........**  **Primary nurses..............** | **Comments** |
| --- | --- |
| **Nurse** |  |
| *1. Nursing station. Nurses were doing the paperwork. I can hear that the noise from the working printer. They were so busy.*  *2…*  *3…* |  |
| **Patient and/or family member** |  |
| *1…*  *2. Corridor 1. A patient was walking with the handrail. The family member is on her side.*  *3…* |  |
| **Nurse-Patient-Family member** |  |
| *1....*  *2....*  *3. Patient room. The nurse, the patient and his wife laughed and even had a small talk.* | *3. It appeared that the atmosphere was friendly, and the nurse was relaxed while administering the treatment.* |
| **Reflections:**   - *I feel their relationship is so important for patient participation.* | |
| **Notes for future observation:**   - ...   **Notes for future interview:**   - *To ask nurses the way they interact with patients in different relationships.* | |

The first row shows a system for labelling observations, including its number, date, time, and the primary nurses who helped with recruitment. Information about sites and individuals was recorded in the relevant sections of the fieldnotes. Subsequent rows detailed descriptions related to nurses, patients, family members, and interactions among them. My comments were added separately from the descriptive narrative in the second column. Reflections on my overall views, feelings, or thoughts were recorded next. The final row contained notes for planning future observations and interviews. This approach aimed to create comprehensive fieldnotes that were aligned with the research objectives, inclusive, and detailed, rather than merely summarising the observations.

Reference:

Nicholls CM, Mills L and Kotecha M. Observation. In: Ritchie J, Lewis J, Nicholls CM and Ormston R (eds) *Qualitative research practice: a guide for social science students and researchers*. 2nd ed. Los Angeles, California: SAGE LTD, 2014, pp.243-265.

# 2.2 Interview guides

The interview guides below were structured for the whole PhD project. The sections with emphasis added were mostly relevant to this manuscript.

## 2.2.1 Nurse interview guide

1) Could you please tell me about your understandings of PP in nursing care?

- How do you define PP in nursing care?
- **In your experience, how do patients typically participate or not participate in their care?**
- What aspects of nursing care do you believe patients should actively participate in?
- What’s your attitudes towards PP in nursing care?
- **What role do family members play during PP?**

2) In your experience as a nurse in this ward (Neurology Department), can you tell me some examples of PP in nursing care?

- Can you describe a situation where a patient actively participated in their care?
- Have you ever encouraged patients to care for themselves? Please tell me more details.
- Have you ever shared information or knowledge with patients? Please tell me more details.
- **Have you ever involved patients in decision making? If yes, please tell me more details. If not, could you tell me the reasons?**
- Have you encountered patients who were reluctant or unable to participate? Why?

3) From your perspective, are there any factors facilitating or hindering PP?

- What factors help encourage patients to participate in their care? Could you provide some examples?
- What challenges or barriers do you face when trying to involve patients in their care?
- Are there any other factors that do you think influence PP, e.g., hospital regulations, workload, ward culture?

4) What are your suggestions to promote PP?

- In your opinion, what could be done to enhance PP in nursing care?
- What support or training do nurses need to better facilitate PP?

## 2.2.2 Patient interview guide

1) Could you please tell me your experience of admission to the Neurology Department?

- Please can you describe the scene when you arrived at this unit? Which staff members (nurses or doctors) was the first to approach to you? What did nurses do for you?

2) Please can you share some of your experience during the hospitalisation? (What does PP in nursing care mean to you?)

- In what ways do you participate in your own care?
- **Can you describe a situation where you were actively involved in your care? How did it make you feel?**
- Could you please tell me some of your experience of interacting with nurses? Do you feel that nurses encourage you to participate? Why or why not?
- **Do you feel that your voice is heard? How do you communicate your needs and preferences to nurses?**
- **What role do your family members play in your care?** Do you have any family members here caring for you? If yes, what kind of care do they provide for you? Do you prefer nurses to do these? If no, please can you share the experience of self-caring? Do you want more care from nurses or family members?

3) What factors influence you to participate?

- What helps you feel more engaged and involved in your care?
- Have you ever wanted to participate more in your care but felt unable to? What prevented you from doing so?

4) Are there any suggestions for improvement in your participation?

- What do you think nurses could do to better support your participation?
- What changes in the hospital would help you feel more included in your care?

5) At last, could you share one thing during your hospital stay that has impressed you the most so far?

## 2.2.3 Family member interview guide

1) Could you please tell me your experience about the patient’s admission?

2) What does PP in nursing care mean to you as a family member?

- In what ways do you think patients should be involved in their own care?
- Can you describe how your xx (e.g., mom, husband) participates in their care?
- **Could you please tell me what you often do when you stay bedside?**
- **In what ways do you support or assist them in being involved in their care?**
- **How do nurses involve you in the care process?**

3) What factors influence you and your xx to participate?

- What challenges do you or your xx face in participating in nursing care decisions?
- Have you noticed any challenges they face in participating?
- What helps make it easier for you and/or your xx to participate in nursing care?

4) Are there any suggestions for improvement in your participation?

- What could nurses or hospitals do to better support you and your xx in participation?
- What kind of resources or guidance do you want to receive during your participation in nursing care?

5) At last, could you share one thing during your hospital stay that has impressed you the most so far?

# Supplementary 3. Analytic process using reflexive thematic analysis

The following tables and figures present the detailed coding processes from my entire PhD study, incorporating findings around PP in decision making discussed in this paper.

The coding process was complex as the broader PhD study has a large dataset with multiple perspectives collected by different methods. To show the stepwise coding process clearly, I first introduce them in four stages according to the important turning points in analytic progress, see **Table 3.1**. After that, I situate them in the six phases of reflexive TA (see **Figure 3.2**). A note is that all themes developed in the analytic process were tentative and provisional. There were more than three versions of themes, but I picked them to assist with the description of my analytic trajectory.

**Table 3.1 Four main stages of coding process**

| Stages | Codes group in NVivo | Themes |
| --- | --- | --- |
| Stage one | - observation group - nurse interview group - patient interview group - family member interview group - *unallocated group* | Theme version 1 (see Table 3.2) |
| Stage two | - Nurse group - Patient and family member (PF) group - *unallocated group* |  |
| Stage three | - Theme groups for the entire dataset | Theme version 2 (see Figure 3.1) |
| Stage four | - Theme groups for the entire dataset | Theme version 3 (final version, see Figure 3.1) |

**Table 3.2 Themes version 1**

| Nurse group | Patient and family member group |
| --- | --- |
| - Active PP in fundamentals of care - Encouraging collaboration - Respecting patient autonomy - Dealing with uncooperative patients’ behaviours | - Maintaining routine - Regaining health - Information exchange - Shared responsibility |

**Figure 3.1 The development of theme version 2 to theme version 3**


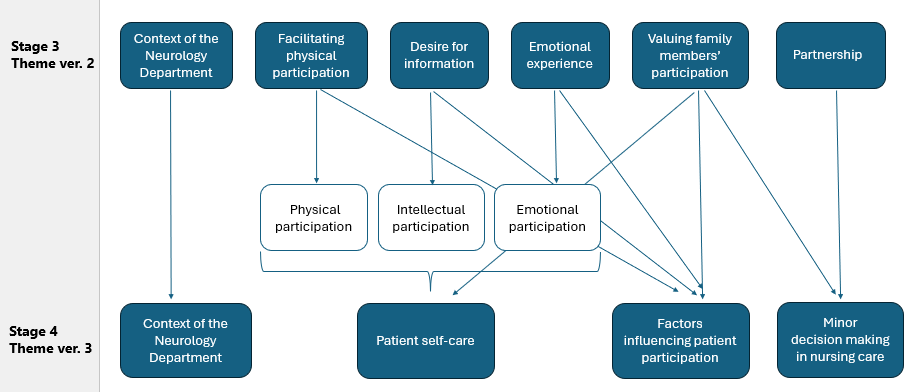


**Figure 3.2 The coding process in six phases of reflexive thematic analysis**


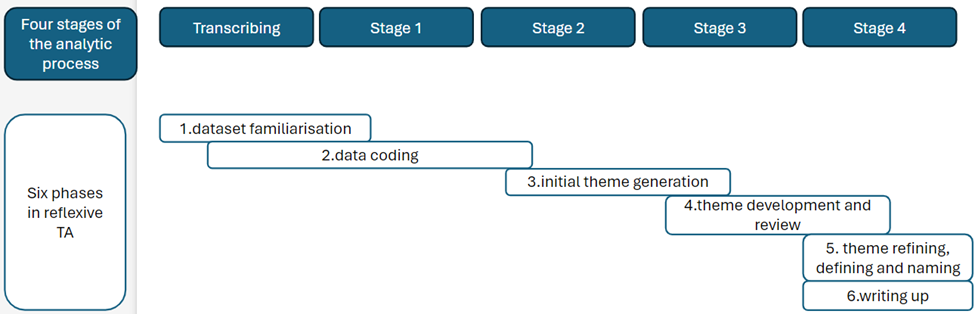

Supplement: Supplementary file 1 — Appendices S1–S3: jan70236‐sup‐0001‐AppendicesS1‐S3.docx. [file JAN-82-6401-s001.docx]
